# Antiarrhythmic Drug Therapy in Arrhythmogenic Right Ventricular Cardiomyopathy

**DOI:** 10.3390/biomedicines11041213

**Published:** 2023-04-19

**Authors:** Sean P. Gaine, Hugh Calkins

**Affiliations:** 1 Department of Medicine, Johns Hopkins University School of Medicine, Baltimore, MD 21287, USA; 2 The Hugh Calkins, Marvin H. Weiner and Jacque J. Bernstein Cardiac Arrhythmia Center, Department of Cardiology, Johns Hopkins University School of Medicine, Baltimore, MD 21287, USA

**Keywords:** ARVC, antiarrhythmic, arrhythmia, flecainide, amiodarone, sotalol, ICD

## Abstract

Arrhythmogenic right ventricular cardiomyopathy (ARVC) is a heritable progressive myocardial disorder that predisposes patients to ventricular arrhythmias and sudden cardiac death. Antiarrhythmic medications have an important role in reducing the frequency of ventricular arrhythmias and the morbidity associated with recurrent implantable cardioverter-defibrillator (ICD) shocks. Although several studies have examined the use of antiarrhythmic drugs in ARVC, these have been mostly retrospective in nature and inconsistent in their methodology, patient population and endpoints. Thus, current prescribing practices are largely based on expert opinion and extrapolation from other diseases. Herein, we discuss the major studies of the use of antiarrhythmics in ARVC, present the current approach employed at the Johns Hopkins Hospital and identify areas where further research is needed. Most notably, there is a great need for high-quality studies with consistent methodology and randomized controlled trial data into the use of antiarrhythmic drugs in ARVC. This would improve management of the condition and ensure antiarrhythmic prescribing is based on robust evidence.

## 1. Introduction

Arrhythmogenic right ventricular cardiomyopathy (ARVC) is a heritable myocardial disorder that is characterized by the progressive replacement of ventricular myocardium with fibrofatty scar tissue, which predisposes patients to ventricular arrhythmias and sudden cardiac death (SCD) [1]. ARVC predominantly affects the right ventricle and is a major cause of SCD in young people and athletes. Treatment of the condition has focused on slowing disease progression, reducing the burden of arrhythmias and the prevention of SCD. Available therapies comprise lifestyle changes, implantable cardioverter-defibrillator (ICD) placement, catheter ablation and the use of antiarrhythmic medications [2]. Antiarrhythmic drugs (AAD) are important adjuncts to ICD placement and catheter ablation in reducing the morbidity associated with ventricular arrhythmias and ICD shocks in patients with refractory arrhythmias. Catheter ablation can be effective in treating malignant arrhythmias in the short-term, but given the progressive nature of the condition, ventricular arrhythmias often recur, necessitating the use of single or combination adjunctive drug therapy to suppress arrhythmias [3]. The aims for this review are threefold: reviewing the current literature and evidence for AAD use in ARVC, detailing the current practice of AAD therapy at Johns Hopkins Hospital based on over 25 years of clinical experience and identifying areas where further research is needed.

## 2. Overview/Pathogenesis

ARVC accounts for approximately 11% of cases of SCD in young adults and is present in the general adult population in approximately 1 in 2000 to 1 in 5000 people [4]. Genetic mutations in desmosomal proteins, which play an integral role in cell-to-cell adhesion, are strongly implicated in the pathogenesis of ARVC and up to 60% of patients with ARVC have pathogenic or likely pathogenic variants in genes encoding the cardiac desmosome [5]. The condition demonstrates variable penetrance and phenotypic expression, but is typically inherited in an autosomal dominant fashion. Patients most commonly present with palpitations or exercise-induced syncope in between the second and fourth decade of life, although SCD may be the first manifestation of the condition [6]. The estimated overall mortality is variously reported among the studies, ranging from 0.08 to 3.6% per year [7,8,9]. Patients with ARVC are risk stratified based on the severity of their arrhythmias and ventricular dysfunction. Prior cardiac arrest due to ventricular fibrillation (VF) and sustained ventricular tachycardia (VT) are the most important predictors of poor outcomes; however, other major risk factors for life-threatening malignant events include unexplained syncope, non-sustained VT (NSVT) and systolic dysfunction of the right and/or left ventricle [2].

The proarrhythmogenic nature of ARVC is complex and involves a number of different mechanisms. Abnormal signal transduction and macro-reentry circuits created by the deposition of fibrofatty scar tissue may lead to the generation of malignant ventricular arrhythmias [10]. Additionally, the interplay between desmosomes, voltage-gated sodium channels and gap junction proteins at the intercalated disc have been implicated in abnormal cell signal transduction and arrhythmogenesis. Animal model studies have shown that a loss of expression of desmosomal proteins may affect the integrity and function of voltage-gated sodium channels. For instance, plakophilin-2 (PKP-2) mutations, which are the most common desmosomal protein variant detected in ARVC, have been shown in murine models to lead to a reduction in INa amplitude. These models have shown increased reentrant activity and significantly decreased conduction velocity in the absence of PKP2, when compared to controls [11].

The proteins involved in intracellular calcium regulation have also been implicated in ARVC. PKP2, for instance, plays a significant role in maintaining the transcription of genes involved in intracellular calcium cycling, such as Ryr2, Cacna1c and Trdn. The loss of PKP2 expression has been shown in murine models to reduce expression of these important genes. Additionally, these models have demonstrated reduced serum calsequestrin-2 levels, a protein that acts as a negative regulator of RyR2 openings [12]. A combination of these factors leads to disruption of intracellular calcium homeostasis [13,14]. Flecainide, which has demonstrated efficacy in catecholaminergic polymorphic ventricular tachycardia (CPVT) and ARVC, is thought to limit the outflow of calcium through the RyR2 channel. This is thought to be one mechanism by which it may suppress ventricular arrhythmias in these conditions [15].

Interestingly, seasonal and circadian variations in arrhythmia burden have been observed in retrospective studies in ARVC. Arrhythmic events have been found to occur with greater frequency in the afternoon and during the winter. It has been proposed that alterations in circadian-regulated sympathetic tone might explain the spike seen in the late afternoon. The increased incidence of infections along with a hemodynamic and sympathetic response to cooler temperatures might explain the higher frequency of arrhythmias during winter [16].

## 3. Indications/Guidelines

The goal of management for ARVC patients is to reduce the risk of SCD, delay disease progression and improve quality of life by reducing arrhythmia burden and heart failure symptoms.

All patients with definite ARVC should be assessed for ICD implantation, as they provide life-saving protection against SCD from arrhythmias. A large observational study by Corrado et al., in 2003 studied the impact of ICD for the prevention of SCD and found that of the 132 patients studied, 24% experienced VF that would likely have been fatal in the absence of the device [17]. The current guidelines recommend their use based on a risk assessment of SCD. Those with prior hemodynamically unstable sustained VT, VF or severe systolic dysfunction of RV, LV or both receive a class I recommendation. ICD should also be considered in those without the above-mentioned indications, but with factors such as inducibility to VT at electrophysiology (EP) study, episodes of NSVT and syncope suspected to be of arrhythmic origin after assessing the risk of SCD. However, they are not recommended as prophylaxis for healthy gene carriers or asymptomatic patients with no risk factors [2,18].

Catheter ablation is another important therapeutic intervention for patients with episodes of sustained VT. It is recommended in patients with recurrent VT or ICD interventions despite a trial of antiarrhythmic therapy. Generally, a combined endocardial/epicardial approach should be considered, particularly if an endocardial approach has failed [19]. However, it is important to note that ablation is not curative and due to the progressive nature of the disease, recurrence rates of VT following endocardial ablation are reported to be up to 50–75% over 3 years [3,20]. Thus, antiarrhythmic drug therapy remains integral in the prevention of symptomatic ventricular arrhythmias, however, importantly, they have not been shown to reduce the risk of SCD in ARVC [17].

In patients with definite ARVC, a beta-blockade (typically long-acting, cardio-selective such as metoprolol) is the recommended first-line in patients with ARVC and recurrent VT, appropriate or inappropriate ICD interventions. Their use should also be considered in all patients with ARVC, irrespective of arrhythmia, but are not recommended for use in healthy gene carriers. The use of beta-blockers in ARVC is based on the observation that beta-blockers lower the risk of SCD in a variety of patients with structural heart disease, prior MI and heart failure. Moreover, they are effective in preventing effort-induced ventricular arrhythmias and may slow disease progression by reducing adrenergic stimulation from exercise and RV wall stress [21].

The 2015 International Task Force Consensus Statement on ARVC recommends AAD use as an adjunct to ICD therapy in ARVC patients with frequent appropriate device discharges and provides class IIa recommendation for AAD for patients with frequent symptomatic premature ventricular contractions (PVCs) or NSVT. A class IIb recommendation is provided for AAD use as an adjunct to catheter ablation in patients without ICD but with recurrent hemodynamically stable VT [2].

The HRS (Heart Rhythm Society) 2019 expert consensus statement on arrhythmogenic cardiomyopathy (ACM), which incorporates ARVC along with a spectrum of genetic, systemic, infectious and inflammatory disorders (sarcoid, amyloid, Chagas disease), provides a class IIb recommendation for amiodarone and sotalol in individuals with ACM for the control of arrhythmic symptoms or reduction of ICD shocks. Flecainide in combination with beta-blockers and in the absence of other antiarrhythmic drugs receives a class IIb recommendation in individuals with ARVC, and ICD and preserved LV and RV function for control of ventricular arrhythmias refractory to other therapies [18]. In the absence of prospective and randomized trials on antiarrhythmic therapy in ARVC or systematic comparison of specific treatment strategies, the choice of drug therapy is based on limited evidence from retrospective data, extrapolation from other diseases and expert opinion.

## 4. AAD Drug Trials/Current Evidence

Sotalol, amiodarone and flecainide are the most utilized AADs in clinical practice; less commonly, mexiletine and others have been used in patients with refractory arrhythmias despite AAD therapy and catheter ablation.

Sotalol is a class III AAD used for both atrial and ventricular arrhythmias. It is a racemic mixture of d and I isomers in an approximate 1:1 ratio, which exert different antiarrhythmic effects. It is generally well tolerated, but can cause bradycardia and has been associated with QTc prolongation and torsades de pointes [22]. It is used to treat both atrial and ventricular arrhythmias and in patients with ARVC who have persistent ventricular arrhythmias, sotalol has traditionally been the first-line AAD of choice.

Amiodarone is also classified as a class III AAD and has complex electrophysiological properties. It prolongs the duration of the action potential and the refractory period primarily mediated through inhibition of IKr. It also exhibits beta-blocker activity, inhibits inactivated sodium channels, and has some class IV effects by blocking L-type calcium channels. It has been hypothesized that amiodarone, a highly lipophilic compound, may deposit at higher concentrations with epicardial fat and cardiac tissues in patients with ARVC due to its unique histologic characteristics, such as fibrofatty replacement of myocardium [23]. Its use is limited, particularly in younger patients, by its high incidence of adverse effects (pulmonary, cardiac, hepatic, ocular, thyroid and dermatologic) with long-term use [24].

Flecainide is a class 1c AAD that produces a selective blockade of cardiac fast inward sodium current, resulting in slowed conduction and prolonged refractoriness. It also inhibits the opening of IKr, resulting in prolonged action potential duration in ventricular and atrial muscle fibers but shortened APD in Purkinje fibers, owing to its sodium channel blockade. Flecainide has also been shown to block the RYR opening, thereby reducing spontaneous sarcoplasmic reticulum calcium release [25]. This RYR blockade is believed to contribute to its particular efficacy in CPVT (catecholaminergic polymorphic ventricular tachycardia), most commonly caused by mutations in the RYR [26]. It can be an effective agent for both ventricular and atrial arrhythmias; however, its use has been limited by significant proarrhythmic effects, particularly in the post-MI population demonstrated in the CAST trial and evidence from animal models that have suggested it may be pro-arrhythmic in the setting of PKP2 haploinsuffiency—the most common genetic mutation observed in ARVC [27,28].

## 5. Major Trials on AAD Therapy in ARVC

The first major study to investigate AAD use in ARVC was performed by Wichter et al., 1992 [29]. This prospective and retrospective analysis found sotalol to be the most efficacious agent in suppressing ventricular arrhythmias in ARVC. In the study, programmed ventricular stimulation (PVS) was performed on 81 patients with biopsy-proven (54.7%) or highly suspected ARVC. In 42 patients, VT was inducible by PVS and in the remaining 39, VT was non-inducible. A series of drug trials were subsequently performed using a variety of AADs. Among the patients with inducible VT, a complete response was defined as no inducibility on PVS, and a partial response was defined as more difficult inducibility (≥2 steps in basic drive). Among those with non-inducible VT, the response was determined by 48-h Holter monitor and symptom-limited treadmill testing. Complete response in patients with non-inducible VT was defined as 100% suppression of NSVT and ventricular runs, and partial response was defined as 100% suppression of NSVT and >70% reduction in ventricular runs, PVCs and ventricular couplets. The trial found sotalol to be highly effective in partial or complete suppression of inducible VT with a 68.4% response rate, as well as an 82.5% response in patients with non-inducible VT. It also concluded that amiodarone was not similarly effective in patients with inducible and non-inducible VT (15.4% and 25% response, respectively). Additionally, a poor response to class 1 agents was observed, with only 5.6% of the inducible VT and 0% of non-inducible VT cohort responding to class 1a or b agents. A 5.3% response to class 1c agents was seen in inducible VT and a 17% response in non-inducible VT. Additionally, a total of 37 patients (26 inducible, 11 non-inducible VT) were treated with combination therapy after failure of response to single agents. A total of 4 out of the 26 patients (15.4%) with inducible VT responded to combinations of a class 1c agent and sotalol (2/10) or amiodarone (2/4). Just 1 out of 11 patients with non-inducible VT responded to a combination of a class 1c agent and amiodarone (1/2).

The next major trial was published by Marcus et al., in 2009: a retrospective study that investigated patients from the North American ARVC Registry. The study enrolled 95 individuals with definite ARVC and ICD placement with frequent PVCs (at least 1000 during a 24-h Holter study). They analyzed the effect of AAD on these 95 patients and followed them for a median period of 480 ± 389 days. Contrasted to the earlier study by Wichter et al., this study came to the opposite conclusion, that sotalol was ineffective in preventing ventricular arrhythmia and ICD shocks, while those on amiodarone had a significantly lower rate of clinically relevant ventricular arrhythmia [30]. Over the course of the study, the patients were treated with a β-blocker (n = 58), sotalol (n = 38) or amiodarone (n = 10). In this series, amiodarone was the only AAD associated with a reduction in major ventricular arrhythmia (MVA) HR: 0.25, 95% CI: 0.07–0.95 *p* = 0.0041), although the number of patients taking amiodarone was notably small. In contrast to the efficacy demonstrated in Wichter et al., sotalol was associated with an increased risk of clinically relevant VAs HR: 2.55, 95% CI: 1.02–6.39 *p* = 0.045, increased risk of ICD shock HR 2.16, 95% CI: 1.15–4.07 *p* = 0.017 and non-significantly associated with increased risk of first ICD shock HR 1.59, 95% CI (0.69–3.63, *p* = 0.28). Beta-blockers were not significantly more or less likely to experience MVA or first arrhythmia, but demonstrated a non-significant reduction in total ICD shocks HR 0.54, 95% CI (0.25–1.18, *p* = 0.12).

Subsequently, in 2017, an important case series by Erkamov et al., described the clinical course of 8 patients with definite ARVC and recurrent ventricular arrhythmias despite sotalol or metoprolol therapy. Of the 8 patients in the series, 6 demonstrated excellent arrhythmia control after the initiation of combination therapy with flecainide and sotalol (4/5) or metoprolol (2/3) without significant adverse effects [31,32].

A more recent retrospective analysis by Cappelletto et al., in 2021 analyzed the effect of β-blockers, amiodarone and sotalol and the risk of sudden cardiac death or recurrent MVA in 123 patients with definite ARVC. The patients were enrolled from the Trieste Heart Muscle Disease Registry and were followed for a median period of 132 months. In contrast to the Marcus et al., study, just 36% of these patients had an ICD. The primary endpoint was a composite of sudden cardiac death, MVA and appropriate ICD shock. The study found that only beta-blockers at >50% of the target dose were associated with a significant reduction in the primary endpoint HR: 0.10, CI: 0.02–0.46 *p* = 0.004. The study notably did not show a significant reduction with either sotalol HR 1.55, 95% CI: 0.71–3.4 *p* = 0.269 or amiodarone HR 0.7, 95% CI: 0.25–1.93 *p* = 0.492. None of the tested AADs were associated with a lower risk of the secondary outcome of SCD/time to the first MVA [33].

Lastly, another retrospective analysis by Rolland et al., published in 2022 investigated 100 ARVC patients (86 definite, 14 borderline) who received flecainide with beta-blockers for the treatment of symptomatic PVCs, VT and NSVT. The patients were followed for a median of 47 months. In patients with a 24-h Holter available (46/100 patients), a significant decrease in 24-h PVC burden was observed with flecainide [median 2370 (IQR 1572–3400) before vs. 415 (97–730) after. Additionally, 33/100 underwent PVS before and after the introduction of flecainide. In these patients, 94% demonstrated a positive PVS before the introduction of flecainide compared with 40% while on treatment, indicating a 58% efficacy rate. Similar results were obtained after excluding patients without a definite ARVC diagnosis. Importantly, flecainide was well tolerated overall with a 10% discontinuation rate: 6 due to lack of efficacy, 1 due to new atrial fibrillation and 3 with other non-specific side effects [34]. The superior efficacy of flecainide observed in this study compared with previous studies, such as Wichter et al., may have been due to a synergic effect of flecainide with beta-blockers. In addition, 20% of patients underwent VT ablation during follow-up, which may have resulted in overestimated efficacy.

Scant data exist on the use of other agents in ARVC. In Wichter et al., 19 patients with non-inducible VT and 20 patients with inducible VT received propafenone. However, the efficacy for suppression of inducible VT was poor for the class 1c antiarrhythmics, as described above. A single case report details the efficacy of propafenone in the termination of VT with intravenous propafenone [35]. Another case report documents QRS widening in a child with ARVC following the initiation of oral propafenone [36].

Similarly scarce literature exists for the use of class 1a and 1b agents in ARVC. Only one of 28 tests performed with class 1a or 1b drugs was effective in suppressing inducible VT. Disopyramide, a class 1a agent, demonstrated partial suppression of VT in 1 of 18 patients studied. One case report describes the combined use of oral disopyramide and mexiletine to suppress recurrent VT in patient with ARVC [37]. Mexiletine, a class 1b agent, was not effective in the Wichter et al. study. However, it has been used as a monotherapy in one case report of ARVC in a neonate in combination with amiodarone to terminate frequent PVCs [38]

## 6. Current Practice at the Johns Hopkins Hospital

**Figure 1:**
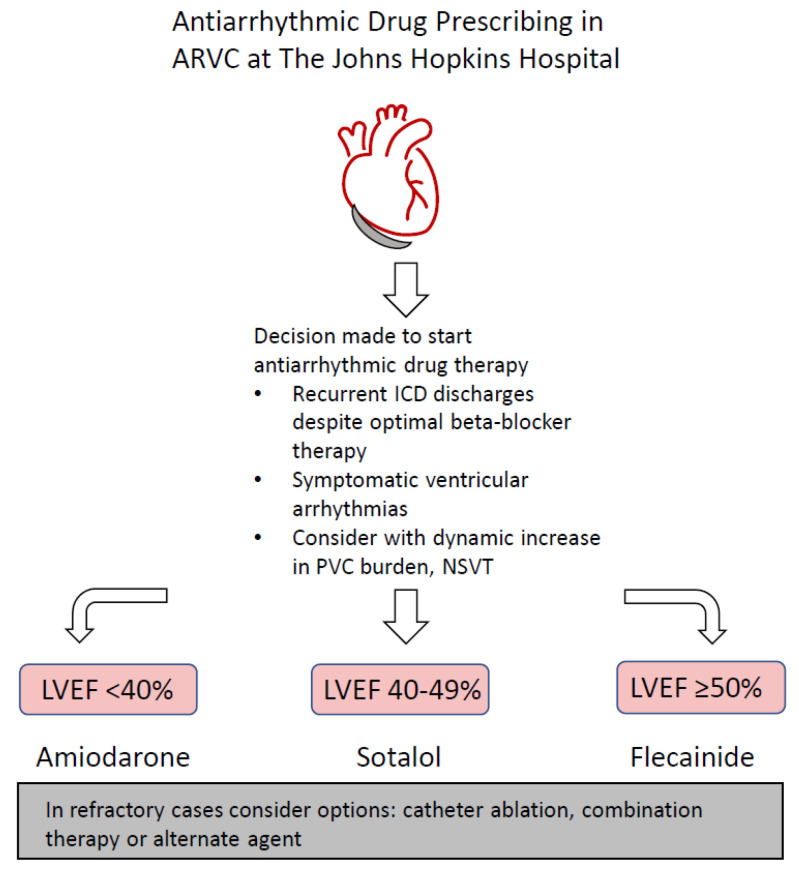
Antiarrhythmic Drug Prescribing in arrhythmogenic right ventricular cardiomyopathy (ARVC) at The Johns Hopkins Hospital.

Given the marked lack of high-quality evidence and inconsistent and conflicting results, our current approach to AAD therapy in ARVC at The Johns Hopkins Hospital is guided by 25 years of clinical experience in treating patients with this condition. AADs are prescribed for patients with the aforementioned indications of recurrent ICD shocks or symptomatic major ventricular arrhythmias. Additionally, patients with a high or dynamic increase in PVC burden also warrant consideration. A recent 2022 retrospective cohort study by Gasperetti et al., demonstrated that the presence of NSVT and/or PVC spikes at a follow-up Holter examination were associated with sustained VA in the following 12 months [39]. A dynamic risk estimation from annual Holter monitor data can prompt the initiation or titration of AAD in an effort to reduce the subsequent risk of major VAs. When the decision is made to initiate AAD therapy, left ventricular ejection fraction (LVEF) < 40% is often used to guide treatment (see Figure 1). In patients with reduced LVEF (<40%), flecainide is generally avoided in favor of amiodarone. In patients with normal EF (>50%), flecainide is often used as the first agent of choice, while sotalol is favored in patients with mid-range EF (40–49%). Combination therapy or additional agents, such as mexiletine, are occasionally used in patients with refractory VAs to the medications above.

## 7. Discussion

While a number of agents have been used to control symptomatic ventricular arrhythmias in ARVC, there remains a lack of consensus about the optimal AAD strategy for the condition. The available evidence is heavily limited by the fact that studies have been mostly retrospective with key differences in their study cohort, methodology and endpoints (Table 1), which makes comparison very challenging. One is unable to draw any meaningful conclusions from the available studies given the striking lack of consistency and conflicting findings between the studies.

For instance, the North American ARVC Registry cohort studied by Marcus et al., may have been of higher risk given that all 95 patients had definite ARVC with implanted ICDs. In contrast, in Cappelletto et al., a minority of patients (36%) had ICD implanted at the baseline and Wichter et al., was conducted in the pre-ICD era. The studies also varied in their methods of medication selection. In Wichter et al., serial programmed stimulation was used in contrast to Cappelletto et al. [33] and Marcus et al. [30], in which treatment decisions were guided by provider preference [40]. Moreover, higher doses of sotalol were used in the cohort investigated by Wichter et al. [29] when compared with the North American Marcus et al., study (320–640 mg/day and 160–320 mg/day, respectively). Importantly, flecainide was not examined in either the North American ARVC Registry, or Trieste Heart Muscle Disease Registry studies, but demonstrated good efficacy and tolerability in Rolland et al. [34] and Erkamov et al. [31]

While sotalol had historically been considered the most effective for this indication based on data from the 1992 Wichter et al. [39] study, the Trieste Heart Muscle Disease Registry in Capelletto et al., did not demonstrate any benefit with sotalol, and the North American ARVC Registry studied in Marcus et al., even suggested a negative effect with an increased risk of ICD shock and clinically relevant arrhythmia. In their study, Marcus et al., acknowledge the potential for confounding by indication, where the likelihood of having arrhythmias could affect the administration of sotalol. However, this would imply that individuals with a greater risk of arrhythmias were also more likely to receive sotalol and does not explain why those who received sotalol were at a higher risk for their first clinically significant ventricular arrhythmia.

The 2015 International Task Force Consensus Statement on ARVC suggests that amiodarone alone or in combination with β-blockers is the most effective drug for preventing symptomatic ventricular arrhythmias based on available evidence at the time of its publication. Nevertheless, neither Wichter et al., nor Capelletto et al., demonstrated any significant effect with amiodarone. Importantly, a recent retrospective analysis by Lin et al., in 2022 suggests worse outcomes with amiodarone use in ARVC patients undergoing catheter ablation. The study examined ablation outcomes in ARVC in patients taking and not taking amiodarone and found that those taking amiodarone pre-ablation had a higher incidence of VT recurrence, a longer activation time and a greater area of abnormal electrograms within the RV, despite similar ablation strategy and endpoints [41]. Additionally, amiodarone use is often limited in practice, particularly in younger patients, by its high incidence of adverse effects with long-term use.

More recent evidence from Erkamov et al. [31] and the recent retrospective Rolland et al. [34] study suggests that flecainide is both safe and effective for reducing symptomatic ventricular arrhythmias. While there has been a historic hesitation of using class Ic agents in patients with ventricular dysfunction based on the post-MI population studied in the CAST trial, these studies provide welcome reassurance for its use [28]. Moreover, contrary to what has been suggested in animal models of PKP2 haploinsufficiency, there was good tolerance of flecainide associated with beta-blockers in Rolland et al., and Erkamov et al., with no Brugada-induced ECG pattern or high-degree atrioventricular block observed [29,34]. There remains a dearth of evidence for the use of other agents, such as mexiletine, propafenone or verapamil, in ARVC aside from the data provided in Wichter et al., and isolated case reports.

## 8. Future Directions

It is clear that there is a great need for high-quality, prospective and randomized controlled studies into the use of AADs in ARVC. Decisions on AAD prescribing remain largely based on expert opinion with limited and often conflicting data from the available studies. A randomized double-blind placebo-controlled crossover pilot study in 38 patients with ARVC is currently underway to evaluate the efficacy of flecainide in the reduction of ventricular arrhythmias in ARVC patients with ICDs (ClinicalTrials.gov identifier: NCT03685149). Further similar studies with consistent patient selection, methodology and endpoints are critical for ensuring that AAD prescribing in ARVC is based on robust evidence. Additionally, the circadian and seasonal variability in arrhythmia burden observed in ARVC is noteworthy and might present opportunities for increased monitoring and more aggressive therapy or increased sympathetic blockade during the winter months when the arrhythmia burden is higher. Furthermore, it is unknown whether specific genetic mutations confer better response to certain medications, which provides further opportunities for research into the tailored use of these medications.

## 9. Conclusions

The management of ventricular arrhythmias is an important and challenging aspect of treatment in ARVC. Antiarrhythmic drugs are used to reduce the significant morbidity associated with ventricular arrhythmias and ICD shocks in these patients. However, no single medication nor catheter ablation results in complete arrhythmia control and due to the progressive nature of the disease, ventricular arrhythmias tend to recur over time. There remains a paucity of data to guide the optimal sequence of antiarrhythmic treatment and combination therapy in refractory cases. While there has historically been hesitation in the use of class 1c antiarrhythmic agents in ARVC, more recent studies and anecdotal use of flecainide, in particular, suggest it can be efficacious and safe in controlling ventricular arrhythmias. Further high-quality studies into AAD therapy in ARVC are needed to address these gaps and improve the management of this condition.

## Figures and Tables

**Table 1:** Important studies on antiarrhythmic drugs in arrhythmogenic right ventricular cardiomyopathy (ARVC).

	Wichter et al., (1992) [29]	Marcus et al., (2009) [30]	Erkamov et al., (2017) [31]	Capelletto et al., (2021) [33]	Rolland et al., (2022) [34]
Study population/Design	81 patients. Retrospective and prospective analysis.	95 patients. Retrospective analysis.	Flecainide combination therapy: *n* = 8 Case series.	123 patients. Retrospective analysis.	100 patients. Retrospective analysis.
Cohort characteristic/Diagnosis	Proven or highly suspected. EMB proven (54.7%).	Definite ARVC with ICD (100%).	Definite ARVC (100%).	Definite ARVC (100%). 36% with ICD.	Definite ARVC (86%). Borderline ARVC (14%). PKP2 mutation (38%).
Duration	34 ± 25 months (inducible cohort) 14 ± 13 months (non-inducible cohort).	480+/−389 days.	NA—case series.	132 months range (61–255 months).	47 months (IQR 23–73).
Medications examined:	Multiple: Sotalol, Amiodarone, β-blocker, Verapamil, Class 1a/b (grouped), Class c (grouped), combination therapy.	β-blocker (*n* = 58), Sotalol (*n* = 38), Amiodarone (*n* = 10).	Flecainide & Sotalol (*n* = 5/8), Flecainide & Metoprolol (*n* = 3/8).	β-blocker, Sotalol, Amiodarone.	Flecainide (*n* = 100) with β-blocker (*n* = 91).
Endpoint:	**IVT cohort (42/81):** Complete response: No VT inducibility. Partial response: more difficult to stimulate > 2 steps in basic drive. Failure: unchanged or spontaneous recurrence. **Non-IVT cohort (39/81):** 48-h Holter guided therapy and symptom-limited treadmill testing. Complete response: 100% suppression of sVT, nsVT, v-runs, ≥70% reduction in couplets/PVCs. Partial: 100% sVT, nsVT suppression but with ≥70–99% v-runs suppression and >70% reduction in couplets/PVCs. Failure: <100% sVT +/− nsVT suppression OR <70% v-runs reduction OR <70% couplets/PVC reduction.	Clinically relevant arrhythmia (sustained VT/VF requiring ICD ATP or shock), first clinically relevant arrhythmia or first ICD shock.	Ventricular arrhythmia/ICD-shock-free period.	**Primary endpoint:** Composite of SCD or MVA (sudden cardiac arrest/sustained VT/appropriate ICD intervention) including recurrent MVA in patients >1 MVA. **Secondary endpoint:** Time to first event (SCD/first MVA).	Sustained VA (VT/VF >30 s) or ICD therapy. PVC burden. PVS.
Efficacy/Outcomes:	**IVT** (1) Sotalol response: complete 22/38 (57.9%), partial 4/35, overall 26/38 (68.4%). (2) Amiodarone response: complete 2/13 (15.4%), partial 0/13, overall 2/13 (15.4%). (3) Verapamil response: overall 0/5 (0%) (4) β-blocker response 0/7 (0%) (5) Class 1a/b response: complete 0/18, partial 1/18, overall (5.6%) 6) Class 1c response: complete 1/25 (4%), partial 2/25, overall 3/25 (12%) 7) Combination therapy: Two class 1 drugs: 0/5 (0%) Class 1 + β-blocker: 0/7 (0%) Class 1 + sotalol: complete 2/10, partial 0/10, overall 2/10 (20%) Class 1 + amiodarone: complete 0/4 (0%), partial 2/4, overall 2/4 (50%). **Non-IVT** (1) Sotalol response: complete 23/35 (65.7%), partial 6/35, overall 29/35 (82.8%). (2) Amiodarone response: complete 0/4 (0%), partial 1/4, overall 1/4 (25%). (3) Verapamil response: complete 7/24 (29.2%), partial 5/24, overall 12/24 (50%) (4) β-blocker response: complete 0/7 (0%), partial 2/7, overall 2/7 (28.6%) (5) Class 1a/b response: overall 0/16 (0%) (6) Class 1c response: complete 1/23 (4.4%), partial 3/23, overall 4/23 (17.4%) (7) Combination therapy: Two class 1 drugs: 0/5 (0%) Class 1 + β-blocker: 0/7 (0%) Class 1 + sotalol: overall 0/10 (0%) Class 1 + amiodarone: complete 0/2 (0%), partial 1/2, overall 1/2 (50%).	**Overall VA event rate:** 25% event rate per patient-year. **Clinically relevant arrhythmia:** (1) β-blocker: HR 1.75, 95% CI (0.48–6.37, *p* = 0.40) (2) Sotalol: HR 2.55, 95% CI (1.02–6.39, *p* = 0.045) (3) Amiodarone: HR 0.25, 95% CI (0.07–0.95) **Any ICD shock:** (1) β-blocker: HR 0.54, 95% CI (0.25–1.18, *p* = 0.12) (2) Sotalol: HR 2.16, 95% CI (1.15–4.07, *p* = 0.017) (3) Amiodarone: HR 0 (in all the first events occurred in those not taking amiodarone before they occurred in those taking amiodarone) **First ICD shock:** (1) β-blocker: not associated with reduced risk (2) Sotalol: HR 1.59, 95% CI (0.69–3.63, *p* = 0.28) (3) Amiodarone: HR 0	Case (1) 116 months— sotalol/flecainide Case (2) 38 months—sotalol/flecainide Case (3) 46 months—sotalol/flecainide Case (4) 22 months—sotalol/flecainide Case (5) 18 months—metoprolol/flecainide Case (6) sotalol/flecainide—failure within 2 months Case (7) 24 months—metoprolol/flecainide Case (8) metoprolol/flecainide—failure within 2 months	**Primary endpoint:** β-blocker: HR 0.54, 95% CI (0.13–2.14, *p* = 0.378) β-blocker (≥50% target dose): HR 0.10, 95% CI (0.02–0.46, *p* = 0.004) Sotalol: HR 1.55, 95% CI (0.71–3.4. *p* = 0.269) Amiodarone: HR 0.7, 95% CI (0.25–1.93, *p* = 0.492) **Secondary endpoint:** β-blocker: HR 0.82, 95% CI (0.33–2.05, *p* = 0.669) β-blocker (≥50% target dose): HR 0.42, 95% CI (0.09–1.91, *p* = 0.260) Sotalol: HR 1.37, 95% CI (0.46–4.15. *p* = 0.572) Amiodarone: HR 2.63, 95% CI (0.70–9.86, *p* = 0.151)	**Overall VA event rate:** <5% per patient-year, 25% at 5 years. **PVC burden:** available for 46 patients—decrease in 24-h PVC burden under flecainide [median 2370 (IQR 1572–3400) before vs. 415 (97–730), *p* < 0.0001]. **PVS**: 33 patients had PVS performed before and after the introduction of flecainide. Among them, 94% (*n* = 31) had a positive PVS result off-treatment vs. 40% (*n* = 13) on-treatment (*p* < 0.001).

ARVC—Arrhythmogenic Right Ventricular Cardiomyopathy; EMB—endomyocardial biopsy; ICD—implantable cardioverter-defibrillator; NA—Not available; IVT—inducible ventricular tachycardia, PVC—Premature Ventricular Contraction; PVS—Programmed Ventricular Stimulation; HR—Hazard Ratio; MVA—Major Ventricular Arrhythmia; sVT—Sustained Ventricular Tachycardia; nsVT—Non-Sustained Ventricular Tachycardia; V-runs—Ventricular runs.

## Data Availability

Not applicable.
